# Curricular initiatives that enhance student knowledge and perceptions of sexual and gender minority groups: a critical interpretive synthesis

**Published:** 2016-10-18

**Authors:** Jennifer Desrosiers, Tim Wilkinson, Gillian Abel, Suzanne Pitama

**Affiliations:** 1 Department of Population Health, University of Otago, Christchurch, New Zealand; 2 Faculty of Medicine, University of Otago, Christchurch, New Zealand; 3 Maori/Indigenous Health Institute, University of Otago, Christchurch, New Zealand

## Abstract

**Background:**

There is no accepted best practice for optimizing tertiary student knowledge, perceptions, and skills to care for sexual and gender diverse groups. The objective of this research was to synthesize the relevant literature regarding effective curricular initiatives designed to enhance tertiary level student knowledge, perceptions, and skills to care for sexual and gender diverse populations.

**Methods:**

A modified Critical Interpretive Synthesis using a systematic search strategy was conducted in 2015. This method was chosen to synthesize the relevant qualitative and quantitative literature as it allows for the depth and breadth of information to be captured and new constructs to be illuminated. Databases searched include AMED, CINAHL EBM Reviews, ERIC, Ovid MEDLINE, Ovid Nursing Database, PsychInfo, and Google Scholar.

**Results:**

Thirty-one articles were included in this review. Curricular initiatives ranging from discrete to multimodal approaches have been implemented. Successful initiatives included discrete sessions with time for processing, and multi-modal strategies. Multi-modal approaches that encouraged awareness of one’s lens and privilege in conjunction with facilitated communication seemed the most effective.

**Conclusions:**

The literature is limited to the evaluation of explicit curricula. The wider cultural competence literature offers further insight by highlighting the importance of broad and embedded forces including social influences, the institutional climate, and the implicit, or hidden, curriculum. A combined interpretation of the complementary cultural competence and sexual and gender diversity literature provides a novel understanding of the optimal content and context for the delivery of a successful curricular initiative.

## Introduction

Sexual and gender minority (SGM) populations experience significant health and social inequity compared to the rest of society.^1^ Sexual minority is a term used to describe the diverse and unique populations who identify as gay, lesbian, or bisexual (LGB), are unsure of their sexual orientation, or have had sexual contact with persons of the same sex or both sexes.^2^ Gender minority groups are described as those whose gender identity or expression differs from the sex they were assigned at birth.^3^

SGM populations are more vulnerable to negative health and social exposures and their associated outcomes. For example, SGM populations experience disproportionately high rates of depression, anxiety, substance abuse, and psychological problems.^4^–^6^ In addition, SGM populations are more likely to attempt suicide, run away from home, and experience harassment and violence.^7^–^11^ Gender minority groups are also more likely to experience discrimination within employment, housing, and healthcare situations.^12^,^13^ For example, Lombardi^14^ found that 37% of those who identified as transgender experienced economic discrimination, and 60% experienced harassment or violence.

The vulnerability and inequity faced by SGM populations may precipitate increased contact and greater need from health and social services. Thus, it is critical that health and social service providers act in an equitable manner and respond accordingly to their crucial role in the health and wellbeing of SGM populations. Despite the health and social disparity faced by SGM populations, the literature suggests that many of those working in health and social services are ill-prepared to effectively care for members of SGM groups. For example, prior literature reports that medical students are not adequately prepared with the knowledge or skills to effectively engage with SGM patients in a clinical setting.^15^,^16^ Likewise, Logie, Bridge & Bridge^4^ found that social work students had low self-reported competence when providing care to SGM populations.

Also, students may be exposed to a narrow view of the health and social needs of SGM groups. Van Voorhis and Wagner^17^ reviewed 12 social work journals, and found that only 1% of the articles related to SGM groups were not focused on HIV/AIDS. This narrow research focus fails to acknowledge the broad social and health needs of SGM populations, and can perpetuate further stigma. In addition, this paucity of literature around SGM populations makes it difficult for those working in social services to find further information about this topic.

The literature also suggests that a significant proportion of health, psychology and social work students may have harmful perceptions about SGM groups^18^–^20^. These beliefs hinder their ability to provide equitable care. Nuyen et al.^20^ reported that twenty-seven percent of medical students had observed judgemental behaviours toward SGM patients from physicians, and slightly more than half had observed judgemental behaviours toward SGM patients from their peers, the hidden curriculum at work. Raiz and Saltzburg^21^ found that less than forty percent of social work students expressed outright acceptance of members of the gay and lesbian community and support for their rights while Logie, Bridge & Bridge^4^ found that a high proportion of social workers are biased against SGM groups.

Lack of preparation to equitably care for SGM groups, in conjunction with harmful perceptions, result in significant implications to health and wellbeing of SGM populations.^6^ For example, SGM populations are more likely to experience inadequate assessment, treatment, and preventive care.^22^ Morrison and L’Heureux^9^ also reported ramifications including discriminatory treatment and assessment, misunderstandings resulting in misdiagnosis, and pathologizing.

A variety of tertiary institutions have added sexual and gender minority related curricular content in an attempt to equip future professionals with the appropriate knowledge, perceptions, and skills to equitably address the needs of SGM groups. A range of curricular initiatives have been implemented to prepare students to engage effectively with SGM groups, however, it is not clear which initiatives are most effective. Few curricular strategies have been evaluated, and many of those that have been, have reported conflicting findings about efficacy.

The complementary Cultural Competence literature, which focuses on preparing students with the requisite knowledge, attitudes, and skills to effectively engage in cross-cultural interactions, may offer valuable insight to guide the development and refinement of SGM curricula.^23^–^25^ According to Betancourt^25^, cultural competence curricula should include an integrated triad of knowledge, attitude and skill components. Each of these components is essential to the success of cultural competence training, but is insufficient on its own.

Although the Cultural Competence literature focuses on different populations than the SGM literature, the same fundamental concepts underpin many of the inequities faced by both populations. For example, SGM and cultural minority groups experience organizational, structural, and clinical barriers including inequitable access to resources, power, health care, and legal standing.^26^ In addition, the inequity experienced by SGM and cultural minority groups is produced and maintained by the same structural forces such as privilege, hegemony, and bias which go largely unexamined by society.^27^–^30^ Thus, shared interpretation of the cultural competence and SGM literature may illuminate the shared foundations between these complementary topics, and further highlight the intersectionality of cultural, sexual, and gender identities.

The cultural competence literature also highlights the necessity of a supportive explicit and implicit curriculum. The explicit curriculum is the curriculum that is intentionally taught and often reflected through stated learning objectives. Conversely, the implicit curriculum, sometimes referred to as the “hidden curriculum”, is the set of premises that are unintentionally or subconsciously taught through interactions, role modelling and the climate of the institution.^31^

Curriculum development in relation to SGM groups is still an emerging field, and has been slower to materialise than for cultural competence. Although social attitudes toward SGM populations have evolved over time, much of the foundational literature in the SGM field consists of the earlier work.^32^ By contrast, the cultural competence literature has undergone significant refinement, evaluation, and critique since its inception, which can offer valuable insight to the development and evaluation of SGM curricula. For example, the integration of knowledge, attitude, and skills components in the explicit curriculum and the awareness of the sub-text of the implicit curriculum is absent in a significant portion of the SGM literature, but included in much of the cultural competence literature.

This paper presents a synthesis of the relevant literature that has described and evaluated curricular initiatives designed to optimize student knowledge, attitudes or skills to care for SGM populations. The paper discusses the characteristics of effective educational initiatives, and places and contrasts these within a broader framework of cultural competence. The role of the implicit curriculum and the assumptions that are embedded in the research regarding student knowledge and perceptions of SGM populations are then discussed. The paper concludes with limitations and recommendations for further research.

## Methods

A modified Critical Interpretive Synthesis (CIS) methodology^33^ was used to amalgamate the qualitative and quantitative data regarding curricular interventions to improve student knowledge and perceptions of SGM populations. This method was chosen because it allows for the synthesis of data from both qualitative and quantitative studies in a way that allows the sum to become greater than its parts and new constructs to become illuminated. The CIS process can be iterative, interactive, and dynamic. It also allows searching, sampling, critique, and analysis to happen concurrently.^33^ Although the framework of a CIS can involve selective and purposive sampling, this review has modified the approach to take a more systematic and comprehensive approach for identification and inclusion of relevant literature. Quality appraisal of qualitative research is contentious, and therefore only qualitative and quantitative studies deemed to be fatally flawed have been excluded.^33^ See Appendix 1 for the completed data extraction form.

### Inclusion and Exclusion Criteria

Articles were eligible for inclusion if they described and qualitatively or quantitatively evaluated a curricular intervention designed to address tertiary student knowledge, perceptions, or skills to care for SGM groups. For pragmatic reasons, only studies written in English were eligible for inclusion. Articles from any time period and both qualitative and quantitative papers were eligible for inclusion in an effort to capture the depth and breadth of information.

Studies were excluded if they did not meet the inclusion criteria, if the initiative was not described in sufficient detail, and if the initiative was not formally evaluated. Qualitative papers were deemed fatally flawed and excluded if they did not have a clear research question; the research question, data collection, or analysis was not appropriate for qualitative research; or claims were not supported by sufficient evidence.

### Literature Search Strategy

**Figure 1:**
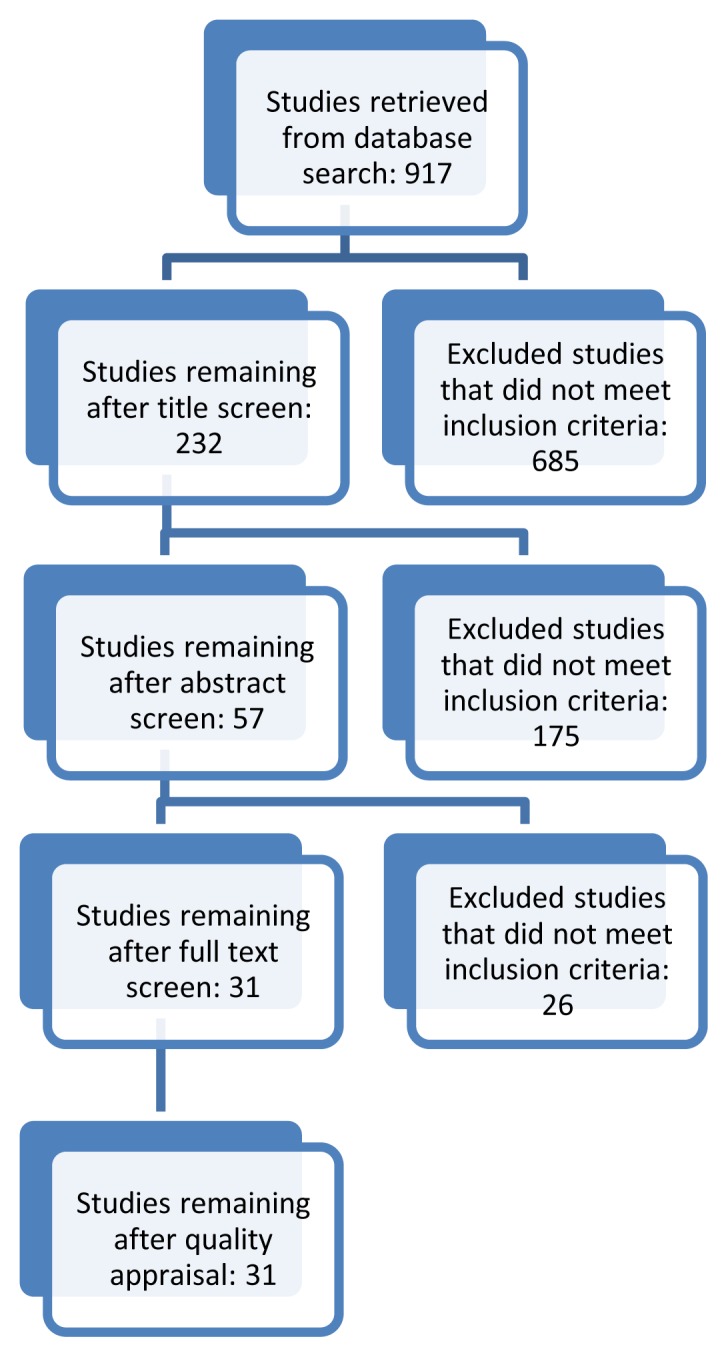
Flow chart of included studies

A literature search was conducted in May 2015 to locate the relevant literature regarding curricular initiatives designed to address student knowledge, perceptions, or skills to care for SMG. No limits were used to restrict the year of publication. Citations and article references were reviewed in order to identify additional articles for potential inclusion. See Figure 1 for a Flow diagram of included and excluded studies.

Resources searched included: AMED, CINAHL EBM Reviews, ERIC, Ovid MEDLINE, Ovid Nursing Database, PsychInfo, and Google Scholar.

Search terms for sexual orientation and gender included: LGBT, lesbian, gay, bisexual, sexual orientation, transgender, transsexual, queer, sexual minority, homosexual, and sexual orientation. Search terms in search engines for education included: medical education, evaluation, and curricul*.

## Results

Thirty-one studies were included in the review. Of the included studies, twenty-two studies had a quantitative component and nine had a qualitative component. Twenty-seven of the included studies were conducted in the USA, one in Israel, one in the UK, and two in Canada. The studies were conducted in a variety of tertiary programs and institutions including medical schools,^14^ dental schools,^1^ social work,^5^ general university programmes,^7^ psychology,^3^ and nursing.^1^

Ten different scales were used to measure student perceptions including: Attitudes Toward Gay Men (ATG), Attitude Toward Lesbians and Gays scale (ATLG), Heterosexual Attitudes Toward Homosexuality scale (HATH), Index of Attitudes Toward Homosexuality (IAH), and Homophobic Behavior of Students Scale (HBSS), Index of Homophobia (IHP), Homosexuality Attitude Scale (HAS), Modern Homonegativity Scale (MHS), Attitudes Regarding Bisexuality Scale (ARBS), adapted Weinberg Homosexuality Scale (WHS), and Riddle Homophobia Scale (RHS).

Of the 31 included studies, 13 used one or more of the different scales to assess student attitudes. See Table 1 for the scales used in each study. Fourteen studies used pre- and post-comparisons, one study compared post-test scores from the intervention group to the control group,^34^ and two studies used student reflections.^35^,^36^ Also, two studies used scales as pre- and post-tests, as well as comparisons between intervention and control groups.^37^,^38^ There is a paucity of research regarding the comparability of the scales, therefore the comparability of the findings will be limited.

A variety of curricular approaches have been implemented in an attempt to provide students with the knowledge, perceptions, and skills required to care for SGM groups. Delivery methods for teaching sessions include discrete sessions, such as lectures, panel sessions, discussions, intergroup dialogues, case vignettes, and movies. Multi-modal strategies include combinations of the aforementioned strategies, as well as coursework paired with clinical exposure, combined research and sexual minority content, and the infusion method which integrates content into substantial portions of coursework.

### Discrete interventions with time for processing

Evaluations of discrete sessions have shown no significant change in knowledge, attitudes, or skills for caring for SGM populations. For example, a one-hour session about transgender populations^34^ showed no significant change to student knowledge. Also, panel sessions have been shown to have little effect on student attitudes.^39^–^41^ Despite the lack of efficacy of discrete interventions such as speaker panels and single teaching sessions, prior literature has suggested that the majority of educational initiatives use these methods.^43^

Although standalone discrete sessions appear to be ineffective, initiatives that paired a discrete session with an opportunity for processing^44^ or informal conversation^45^ showed positive effect on student attitudes toward SGM populations.

### Multimodal sessions

A variety of multi-modal sessions have reported positive shifts in student knowledge about SGM populations including a “Safe Space” program, a cultural humility session, a HEALE curriculum focused on treatment of SGM elders, a three-part intervention, and a SGM Health Issues Immersion Day.^20^,^46^–^49^ The “Safe Space” program content included SGM terminology, bias, stereotypes, coming out, and information about suicide risk, prevention, and resources.^46^ The two-hour cultural humility session consisted of pre-readings, a lecture, a patient as professor panel, and an interactive question and answer opportunity.^47^ The HEALE curriculum included six separate modules: SGM terminology; health disparities; barriers to care; sex and sexuality; the transgender community; and HIV.^48^ The three-part curriculum consisted of a syllabus, patient panel, and small group session focused on case studies.^49^ The SGM Health Immersion Day included lectures, panel presentations, video training modules, and clinical vignettes.^20^

Fourteen multimodal sessions have reported improvements in student perceptions of SGM populations.^35^,^37^,^39^,^45^,^50^–^60^ See Appendix 1 for the details of multimodal strategies designed to improve student perceptions. Multimodal strategies involved a variety of learning opportunities and delivery methods. For example, Bassett & Day^50^ and Levy^58^ integrated SGM content into a range of modules via lectures, activities, discussions and role-plays within the social work program. This method weaved content through the curriculum, which allowed students to continually build on prior learning.

Dessel et al.^54^ conducted an Intergroup Dialogue course, which showed reduction in bias, increased empathy, positive effects on communication across differences, engagement in alliance building, and social justice. This involved students from different, often conflicting, social identity groups with unequal power. A variety of different activities were incorporated, including a fictional scenario where students considered the stereotyping and social stigma that is often directed toward members of sexual minority populations. Results indicate that students maintained their gains in learning, positive attitudes toward sexual and gender diverse groups, and commitment to action after the dialogues ended.^54^,^61^

Ten of the multimodal strategies involved a speaker panel in conjunction with a minimum of one other learning opportunity.^35^,^39^,^45^,^47^,^49^,^51^–^53^,^59^,^60^ Four curricular strategies included videos, in addition to at least one other learning opportunity.^51^,^52^,^60^,^62^ Other strategies included role play,^58^ standardized patient encounters,^35^,^60^ student reflection exercises,^35^,^59^ an imagination exercise where same sex relationships are the norm,^56^ and case studies.^49^,^60^,^62^,^63^

Four of the included studies reported that perceptions changed for some groups but not for others, which indicates that some perceptions may be firmly entrenched and not amenable to change.^37^,^45^,^50^,^59^ For example, Green, Dixon & Gold-Neil^45^ found that panel discussions where members of SGM populations shared their coming out stories only had a positive effect on the attitudes of the female students. This finding was corroborated by Finken^37^ who found that only female students showed reduced anti-gay prejudice at the end of a human sexuality course. Also, Bassett and Day^50^ found that only the students who placed in the midrange level of the Attitudes Toward Lesbians and Gay scale decreased their homophobic and anti-gay attitudes after being taught about SGM populations. Likewise, Rye & Meaney^59^ found that, although their initiative reduced average homonegativity, those who had irrational beliefs about HIV infection experienced increased homonegativity.

Kelley et al.^49^ found that some facets of attitude were less likely to change than others. For example, the absence of change in statements, such as “I believe that homosexuality is immoral” and “I would feel comfortable treating patients I know are LGBT”, suggests that some components of attitude may be more firmly embedded than others.

Six multimodal initiatives included opportunity for skill development and application, including vignettes, standardized patient encounters, and critiquing a video of a patient consultation and providing feedback.^20^,^36^,^49^,^62^,^64^,^65^ These experiences may have improved skills, however only one initiative included an evaluation in this area.^65^ The authors reported that eighty-two percent of participants were able to clearly articulate how to inquire appropriately about the gender of a patient’s sexual partners following three educational sessions, paired with standardized patient encounters.

The initiative by Lambrese & Hunt^63^ also showed an increase in awareness and referral to support services which may reflect improvement to knowledge, attitudes, or skill components. Two other initiatives looked at feelings of preparedness to care for SGM populations, which suggests an improvement in knowledge.^62^,^66^ For example, McGarry, Clarke & Cyr^62^ found that ninety-six percent of participants felt more prepared to care for SGM patients following the session. In summary, a range of different approaches have been taken to optimize student knowledge, perceptions, and skills to care for SGM populations. Successful interventions include discrete sessions with time for processing, and multi-modal teaching, such as integration into the larger curriculum, intergroup dialogue, and the opportunity to apply learning to practice. However, no individual curricular initiatives have included the triad of knowledge, attitude, and skill components. In addition, only one initiative explicitly evaluated a portion of skills. Thus, it is unknown whether these initiatives will have any effect on care provision to SGM groups, and therefore, the health and wellbeing of SGM groups.

## Discussion

Many similarities exist between the cultural competence literature and the SGM literature. However, some aspects of the cultural competence literature have moved beyond the traditional paradigm to establish a more critical consciousness of self and others, as well as a commitment to social justice.^67^ Reflection of self and privilege, awareness of social forces, and a commitment to advocacy were also present in some of the effective SGM initiatives, and may constitute a positive contribution to future initiatives.

The cultural competence literature also provides some additional insight into the necessity of providing well-designed opportunities for practical application of skills.^68^ For example, Kripalani^68^ posits that solely providing knowledge without an opportunity for practical application can fail to acknowledge diversity within groups, emphasize differences, and reinforce stereotyping behaviour. Betancourt^25^ also asserts that teaching “cultural knowledge” in isolation can be more detrimental than helpful. Only six of the SGM initiatives included a skill-based component, and of those, only one initiative evaluated student skills. Thus, a key area for development of the SGM literature will include strategic inclusion of opportunities for practical application of skills.

Many of the SGM initiatives implied that increased knowledge, awareness, or contact will automatically translate to competent care for SGM groups. However, these assumptions are an oversimplification of a complex and multidimensional process. Contact is not always positive, and interactions do not necessarily facilitate insight. Likewise, knowledge acquisition about populations can, if not appropriately conducted, lead to affirmation of existing negative stereotypes and result in uncomfortable encounters.^51^ Thus, simply interacting with a member of the SGM community, or learning a finite set of knowledge items, will not equate to competent care.^23^

Critique of the cultural competence literature by Kumas-Tan et al.^23^ suggests that similar assumptions are embedded within cultural competence training and SGM literature. For example, the cultural competence literature also tends to focus on the *Other* as an object of specialized knowledge. The inherent assumption is that cultural competence will automatically result once sufficient knowledge, awareness, and exposure have been acquired. However, this paradigm does not facilitate any self-reflection on the part of the student about their lens, privilege, or the social structures that contribute to the continuation of dominant discourses.

Focusing on the *Other* also neglects the structural and social forces that contribute to inequity. Therefore, the disparity experienced by SGM populations may be attributed to internal risk factors, biological imperative, pathologized or regarded as an unavoidable consequence of the natural hierarchy instead of as a product of social stigma.^26^,^27^,^69^–^72^ The cultural competence literature complements these findings, and asserts that in many cases, learners are not assessed on their understanding of white privilege, they are only assessed on their understanding of the effects of ethnocentrism and racism on the minority *Other*.^23^

Heteronormative assumptions were also embedded in many of the initiatives and the scales that measured their efficacy. The initiatives were geared toward teaching heterosexual, cisgender students about the sexual and gender minority *Others*. However, prior research has suggested that sexual and gender diversity exist within tertiary student cohorts.^73^ Also, questions on the scales, such as the Index of Attitudes toward Homosexuals^74^ including “I would feel comfortable if a member of my sex made an advance toward me”, and “I would feel comfortable knowing that I was attractive to members of my sex”, shows an embedded assumption that respondents are heterosexual. The scoring system for these scales suggest that comfort with these statements is a reflection of positive attitude, however, this comfort may actually be a reflection of same sex attraction.

The SGM literature included in this review focused on disparity as a result of a marginalized sexual or gender identity in isolation. This siloed approach ignores or oversimplifies the intersectionality of the characteristics of individuals, and does not allow for the illumination of the interplay between identities. The tenets of cultural competence training are very similar to those within SGM education, and therefore, may provide an opportunity for collaboration and co-facilitation to promote greater understanding of intersectionality.

One of the limitations of this article is that it is only a synthesis of explicit curricular initiatives. The influence of the implicit curriculum is highlighted in the cultural competence literature. For example, students are more likely to internalize the unintended messages transmitted by faculty or stakeholders through the quality of interactions, language used, facilitation, preparation, and debriefing than the intended messages from the explicit curriculum.^23^,^75^ Therefore, it is counterintuitive to research the effects of explicit curricular interventions without firstly assessing the perceptions of the people who are delivering the implicit curriculum. Some potential avenues for creating an ideal implicit curriculum have been provided by Kripalani,^68^ and include buy-in from stakeholders and faculty, promoting cultural diversity among medical students and at all levels of the medical school, and development of a cadre of dedicated faculty.

The comparability of findings from each of the included studies is limited by the variation of scales used. In addition, it appeared that many of the staff members that conducted the curricular initiatives also collected student knowledge and perceptions data. Therefore, social response bias may have influenced student responses, presumably in a positive direction. Also, the majority of articles reported on the average change in perception. Very few articles reported on the magnitude of change for specific groups within the overall cohort; those that did found that some groups experienced greater changes than others. Thus, it is possible that these differences also occurred within other initiatives, but were not identified.

Only one of the included articles focused on skill development. Thus, it is unknown whether the majority of initiatives would have any effect on clinical or social outcomes. Although knowledge and perceptions are key components of a curriculum, these components alone are insufficient. Therefore, further development is recommended to find out whether any future initiatives have any effect on care and equity for SGM populations.

### Conclusions

The success of an initiative will depend upon personal characteristics of students, the explicit curriculum, and the implicit curriculum. The literature suggests that an ideal explicit curriculum will include multi-modal teaching strategies that integrate knowledge, attitude, and skill components. The explicit curriculum will also encourage self-reflection and appreciation of structural forces. An optimal implicit curriculum will have support and buy-in from students, staff, and stakeholders. In addition, an ongoing critical reflection of the assumptions, methods, tools, and criteria used to facilitate and evaluate student learning is necessary for the continued growth and refinement of this area.

Further research into faculty and stakeholder perceptions is essential. In addition, the development of institutional support to assist students with the reconciliation of their personal beliefs with the ethical and professional requirements of their future occupation is necessary. Research that evaluates the efficacy of a curricular initiative on provision of care and health and social outcomes is of utmost importance to establish the relevance and utility of this curriculum.

Cultural competence training and SGM educational initiatives have significant fundamental synergies inherent in their concepts and content. Further collaboration and development between these areas could be mutually beneficial, and may enhance student understanding of intersectionality. An exploration of co-facilitated delivery of these topics may further enhance understanding and maximize scarce curricular time.

## Figures and Tables

**Table 1:** Scales and measures used to determine student perceptions of SGM populations

Author	Scale(s) Used	Data Collection
Bassett, J.D. & Day, K.J.	ATLG	Pre- and post-test
Beasley, C. et al.	HATH, IAH, HBSS	Pre- and post-test
Bauman, K.A. & Hale, F.A.	Adapted WHS	Pre- and post-test
Ben-Ari, A.	Modified IHP	Pre- and post-test
Black, Oles, Cramer & Bennett	ATLG	Pre-and post-test
Brondani, M.A. & Patterson, R.	N/A	Student reflections
Dowshen, N. et al.	Survey	Post-test comparison with control
Finkel, Storaasli, Bandele & Schafer	RHS	Self-reported changes via course evaluation
Finken, L.L.	IHP	Pre- and post-test; Intervention group compared to control
Green, Dixon & Gold-Neil	Survey	Pre- and post-test
Grubb et al.	Survey	Pre- and post-test
Grutzeck & Gidycz	Modified IAH and HATH	Pre- and post-test
Guth et al.	IAH, HAS, perceived attitude change	Pre- and post, and follow-up test
Hillman, J. & Martin, R.A.	Homophobia Scale	Pre- and post-test, free text
Johnson, M.H. & Henderson, P.	N/A	Self reflections
Kelley, L. et al.	Survey	Pre- and post-test
Kwon, P. & Hugelshofer, D.S.	ATG, MHS-L, MHS-G, ARBS-T	Intervention group compared to control
Rye, B.J. & Meaney, G.J.	IHP	Pre- and post-test
Wallick, Cambre & Townsend	IAH	Pre-and post, and follow-up tests

## Appendix 1 Completed data extraction form: curricular interventions

**Table:**

Author & Location	Year	Population	Institutional Climate	Objectives	Intervention	Methods	Results	Conclusions
Alonzo, C.A. USA	2014	All students, faculty, and staff at the Health Science Center. N unknown	Not assessed	Correct the lack of knowledge about SGM populations and their perceptions of the healthcare communities’ knowledge base	Safe Space program: one 90 min. workshop re: LGBT terminology, bias, stereotypes, coming out, suicide risk and prevention, resources	Pre and post-training survey to assess baseline knowledge of LGBTQ terminology, the coming out experience, and suicide resources	Significant knowledge improvement re: SGM suicide risk and resources, and feeling prepared to deal with SGM issues	Participants wanted longer sessions to increase discussion times, and have more members of the SGM community present in the presentation
Bassett, J.D. & Day, K.J. USA	2003	1st year Masters in Social Work students N= 48	Not assessed	Examine whether the infusion method reduces homophobic and antigay attitudes	Infusion method - working LGBT content into substantial portions of all coursework	Quasi-experimental design using pre-and post-test using the 20 question ATLG	Reduction in mean scores on ATLG post-test. Most of the highest and lowest scoring individuals unaffected by intervention	Infusion model of instruction may be more effective than panel presentations
Bauman, K.A.; Hale, F.A. USA	1985	Elective course for first year medical students N=16	Not assessed	Develop or find support for a positive and caring attitude toward homosexual people, improve medical knowledge re: risk factors for LGBT patients	11 hours of teaching. Defining terms, caring for homosexual patient, interviewing, medical concerns. Informal discussion with members of SGM community	Attitude questionnaire consisting of 15 items with scaled response adapted from Weinberg 1972	Pre and post seminar attitude scores were higher compared to group that did not attend elective.	Success due to specific, well-defined course objectives, informal and non-threatening learning situations, and the participation of articulate homosexual people
Beasley, C. et al. USA	2012	Students from three college campuses N=176	Not assessed	Examine the influence of an interactive “virtual” gay and lesbian speaker panel on homonegativity	Intervention group: video and virtual panel with LGBT presenters. Control group: information about majoring in psychology.	Experimental design. Compared scores on HATH, IAH, HBSS	Significant reduction in post-test homonegativity for group that participated in the virtual panel.	Private environment where participants can ask questions and interact without social constraints. May limit anxiety and defensive attitudes
Ben-Ari, A. Israel	1998	Third year undergraduate social work students enrolled in elective course N=31	Not assessed	Examine social work students’ attitudes toward homosexuality prior to and following an academic course about homosexuality.	12 hour course involving watching a movie “The Torch Song Trilogy” and meeting a mother and her gay adult son who told their personal stories.	Pre- and post-tests using modified IHP. Free text about perceived change and reasons for change	9 students reported no change, 4 because they were already open to homosexuality. The remaining students reported significant changes in attitude	Students attributed change to a class when a gay son and his mother told their personal stories, as well as to an overall increase in knowledge
Black, Oles, Cramer & Bennett USA	1999	Undergraduate and graduate social work students N=56	Not assessed	Evaluate the effect of professional education sessions students’ attitudes and anticipated professional behaviour (APB) toward gay men and lesbians	4 different professional education sessions	Pre- and post-test using modified ATLG and case vignettes	Attitudes did not change after panel discussions. Different educational interventions did not significantly change APB.	Significant and positive change in APB toward lesbians, but lower pre- and post-test scores overall with APB toward gay men
Brondani, M.A.; Patterson, R. Canada	2011	Dental education year 1 and 2	Not assessed	To expose all students to alternative views of sexuality, challenge their values and beliefs, and celebrate diversity	6 hours including lecture-based seminars, standardized patients, guest panel discussions, poster discussions, and student reflections	Documented student reflections	Positive impact upon students and attitudes as illustrated by their reflections	Include faculty & staff in teaching SGM issues in dental education and better understanding of the implications of such education
Dessel, A.B. et al. USA	2013	Heterosexual undergraduate students N= 54	Not assessed	Explore heterosexual students’ experiences in sexual orientation intergroup dialogue courses	Intergroup dialogue course	Qualitative research design using post dialogue semi-structured interviews	Students reported a greater empathy and understanding of LGB peers, reduction in bias	Students concerned about offending classmates, conflict with classmates about controversial topics
Dowshen, N. et al. USA	2013	Medical students Experimental group N =150 Total N = 204 completed survey	Not assessed	Improve student knowledge, attitudes and skills toward transgender people	One session on transgender health	Post-test knowledge assessment, self-reported changes	No difference in knowledge between intervention and control. Self-reported knowledge improvement re: health, history taking	The amount of time dedicated to teaching about transgender health in medical school needs to be increased
Finkel, Storaasli, Bandele & Schafer	2003	Graduate psychology students N=48	Not assessed	Increase sensitivity toward, knowledge of, and advocacy for LGBT populations and issues that affect them	2 hour Safe Zone diversity program	Post-test self-reported behavioral and attitudinal changes between pre and post intervention	No significant difference between self-reported measures.	More empirical research is needed
Finken, L.L. USA	2008	Intervention group: 147 undergraduate students in a sexuality course. Control: 133 undergraduate students in a child development course	Not assessed	Assess impact of a human sexuality course on students’ attitudes about homosexuality	General information about course, but specifics not clear	Pre and post-test and follow-up scores on IHP for intervention group compared to control	Intervention group reported less homonegativity than the comparison. Only female students showed reduced anti-gay prejudice	Further research needs to find a way to reach male students in the classroom
Green, Dixon & Gold-Neil USA	1993	27 male and 52 female undergraduates.	Not assessed	Alter students attitudes regarding gays, lesbians, and persons with AIDS	Gay/lesbian panel discussion conducted within a university-level human sexuality class	A pretest-posttest to assess student attitudes prior to and directly after the intervention	Change to attitudes of females Males showed no significant change from pretest to posttest	Gay/lesbian panel discussions may only be effective for altering attitudes of females
Greene et al. USA	2014	18 students enrolled in first medical school course	Not assessed	Give students a low-risk opportunity to take a sexual history from a transgender patient	SIM activity taking a sexual history with standardised patients	Qualitative student feedback and observations	Students described the experience as personally and clinically constructive	Some students froze, felt unsure of what to ask and became tearful, all rated the SIM as very useful
Grubb et al. USA	2013	MD and MD/PhD Candidates in the Major Clinical Year N=150	Not assessed	Describe aspects of human sexuality and gender. Identify health care disparities, health needs and strategies to care for SGM patients, examine assumptions	2 hour cultural humility session including pre readings, 1 hour didactic lecture, 1-hour “Patient as Professor” panel followed by an interactive Q&A session	Pre and post test questionnaires were completed.	Resulted in significant increases in medical knowledge and positive shifts in attitudes with respect to LGBT populations	Contextualize panel presentations within the overall curriculum; and vet presenters for potential anger and/or resentment toward health care providers
Grutzeck, S. & Gidycz, C.A. USA	1997	200 Undergraduate students taking introductory psychology	Not assessed	Investigate the effect of a panel presentation on attitudes & behaviors of an undergraduate population, while controlling for context	Panel Discussion and hand-out	Modified IAH and HATH	This type of intervention does not significantly alter intolerant attitudes and behaviors	Most respondents started moderately homophobic and stayed that way, regardless of exposure to the panel discussion
Guth et al. USA	2002	87 undergraduate and graduate students	Not assessed	Assess the influence of training modality on attitudes toward lesbian and gay issues.	Students randomly assigned to one of three workshop modalities (In-Person, Internet, Control). In-person and internet session included reflection activity	Pre-test, post-test, follow up attitudes measured via IAH, HAS, perceived attitude change	Internet-delivered workshop was equally effective at reducing negative attitudes toward sexual diversity as a physically delivered workshop	There was no correlation found between perceived attitude change and IAH, HAS scores
Hardacker, Rubinstein, Hotton & Houlberg USA	2014	Nurses and health-care providers N=848	Not assessed	Curriculum focused on the treatment of LGBT elders	Six-module curriculum entitled Health Education about LGBT (lesbian, gay, bisexual and transgender) Elders (HEALE)	pre-test and post-test knowledge assessed. Free text feedback was also collected	Statistically significant gains in knowledge in each of the six modules	Feedback ranged from: ‘Confused why this information was provided’ to ‘Extremely helpful program’
Hillman, J. & Martin, K.A. USA	2002	Undergraduate students in developmental psychology. N=68	Not assessed	Active learning opportunity to allow students to experience stereotyping and consider the social stigma often directed toward gays and lesbians	Control group: lecture on discrimination & homophobia. Experimental group: imagination exercise re: sex relationships as the norm	Pre and post -test 25 item Homophobia Scale, free text	Decrease in homophobic attitudes. Students increased positive feelings toward minority group members	Authors suggest this method allows for exploration of attitudes in a non-threatening way.
Johnson, M.H. & Henderson, P. UK	2000	Third year preclinical students. N=20,	Not assessed	Self reflection re: perceptions. Acknowledge the way difference is handled in society and the impact that it can have people and on all members of society	Students critically evaluate a video of a patient consultation and write feedback to the consultant to help develop consultation skills, vignettes	Qualitative data collected.	Ignoring or attempting to minimize differences is as bad as discriminating; fear of embarrassment can perpetuate ignorance and prejudice	Academic staff must have group-work training undertake potentially emotionally volatile work on attitude development with students
Kelley, L. et al. USA	2008	Year 2 Postgraduate Medical Students N=75	Not assessed	Increase awareness of students’ assumptions of LGBT people, highlight disparities of health care, underscore the role of physicians in dispelling disparities to optimize LGBT health	3 part intervention involving a syllabus, a 1 hour patient panel and a 1 hour small-group session focused on case studies.	Pre and post intervention surveys assessing knowledge, attitudes and experiences were completed	Increased knowledge re: clinical relevance, access to health care, increased willingness to treat SGM patients	No change in attitude toward comfort treating LGBT patients, immorality of homosexuality which may indicate that attitudes are firmly entrenched by this stage of training
Kwon, P. & Hugelshofer, D.S. USA	2012	Heterosexual undergraduate students N= 186	Not assessed	Compare the effects of a speaker panel presentation versus a control condition in altering attitudes toward sexual minority groups	Speaker panel with control for context factors	Pre and post intervention ATLG, MHS-L, MHS-G, and ARBS tests were conducted	Participants in experimental group showed greater increases in positive attitudes on the ATG, MHS-L, MHS-G, and ARBS-T	The effect sizes in the current study are small, which is to be expected in examining the effects of a one-time brief intervention.
Liddle, B & Stowe, A. USA	2002	Undergraduate human services students	Not assessed	Improve student attitudes toward lesbian, gay, bisexual and transgender people	Presentation by lesbian guest lecturer followed with facilitation by heterosexual instructor	Qualitative feedback gathered	Self-reported attitude change and greater understanding of LGBT issues among some students occurred	Lesbian presenter, opportunity to challenge existing views, opportunity to process with a heterosexual facilitator were important
Lambrese, Hunt USA	2013	Clinicians N= unknkown	Not assessed	Improve knowledge, comfort, and competency of clinicians working with sexual minority teens. self-assess pre-existing attitudes regarding the sexual minority community	60 min. lecture and 90 min. discussion and vignettes using handbook. Prevalence of psychiatric illness in sexual minority teens; practical tips; initiating a conversation about sexual orientation	Attendees completed anonymous, written evaluations which included open ended questions for qualitative assessment.	Self-reported comfort with discussing sexuality with patients increased. 60% of respondents referred a patient to a resource discussed in the course, 70% used the handbook	Clinicians have improved their knowledge of the needs of and resources for sexual minority teens.
Levy USA	2013	Undergraduate social work students. N= 19	Not assessed	Enhance students’ overall awareness and sensitivity to the transgender population with the hope of facilitating increased effectiveness of the students in their future work	Infuse transgender content into 2 undergraduate courses on research methods and cultural competence. Lectures, activities, discussions, and role-plays	Qualitative data based on self-report	Students developed knowledge and attitudes and identified substantial shifts in their understanding about the transgender population	Transgender speakers were important. 3 students were uncomfortable & uncertain re: questions to ask as did not want to offend the speakers
McGarry, Clarke & Cyr USA	2000	37 PGY2 residents in the 3-year General Internal Medicine Residency	Not assessed	Introduce medical residents to LG health care issues, discuss experiences with LG patients, and provide tools to conduct a sensitive interview	3 hour seminar using small group discussion, video, case discussions and didactic sessions.	Survey to assess whether residents felt more prepared to care for lesbian and gay patients	96% felt more prepared to care for LG patients 100% felt it is important to learn about LG health care issues	Residents who felt most uncomfortable treating LG patients prior to the seminar felt more comfortable afterwards
Nuyen et al. USA	2015	Second year medical students N=81	Not assessed	LGBT Health Issues Immersion Day sought to help address the LGBT related educational deficit and to analyze the impact of this educational intervention	Lectures from LGBT resource center, LGBT community member panel Q&A on LGBT health and health care utilization, and video training modules with clinical vignettes	Pre- and post-intervention questionnaires, which were administered using blinded Qualtrics software	Increased self-reported knowledge of LGBT health risks, comfort with LGBT patients & confidence connecting LGBT patients to health services	27.2% and 53.1% of students observed judgmental behaviors towards LGBT patients from physicians and peers respectively
Rye, B.J. & Meaney, G.J. Canada	2009	University students from a range of academic programs N=114 Mean age was 23	Not assessed	Increase awareness of heterosexism to reduce reduce homonegativity	Elective heterosexism awareness workshops involving reflection on personal attitudes. Content included coming out stories, Q&A, myths	Hudson and Ricketts’ (1980) Index of Homophobia was used to measure homonegativity	Intervention participants were significantly less homonegative after the intervention	Participants with irrational beliefs about HIV infection were more homonegative following the workshop
Sack, S. Drabant, B. Perrin, E. USA	2002	Third-year medical students N unknown	Not assessed	Communication about sexuality is central to comprehensive health care; improve student comfort in discussing sexual issues	Didactic presentation, panel discussions, videos, case based scenarios including difficult interviews and standardized patients	Student evaluation	The intervention group showed a slight decrease in homophobic attitudes	Lack of a larger effect is likely due to a high level of baseline acceptance and comfort around issues of sexual orientation in this particular medical school class
Safer, J. Pearce, E. USA	2013	Second-year medical students as part of endocrinology unit N unknown	Not assessed	Demonstrate that a simple content change in a medical school curriculum would increase students’ willingness to care for transgender patients.	Curricular content regarding rigidity of gender identity, treatment regimens, and monitoring requirements for transgender patients was added to endocrinology unit	Anonymous questionnaire administered 1 month before and 1 month after the unit. Shifts in views of 2nd year students compared with views of students not exposed to the curriculum change	Pre-test, 38% of students self-reported anticipated discomfort with caring for transgender patients. Post intervention students reported a 67% drop in discomfort with providing care	Students’ self-reported willingness to care for transgender patients significantly increased
Sequeira, Chakraborti & Panunti USA	2012	Preclinical medical students N= 35	Not assessed	Gauge undergraduate medical students’ SGM related interest and perceptions	4 SGM-related educational sessions including 3 optional 1-hour didactic sessions and 1 standardized patient encounter	Following sessions 1-3, students completed electronic feedback. Responses were analyzed thematically	82% of respondents could clearly articulate how to inquire appropriately about the gender of a patient’s sexual partners	Lack of exposure to SGM content, agreement that SGM material is applicable to students’ work as future physicians
Wallick, Cambre &Townsend USA	1995	186 medical students in their psychiatry clerkship	Not assessed	Explore medical students’ changes in attitude toward homosexuality following mid-year exposure as freshmen to a panel presentation on the topic and, later, following clinical experiences.	3 hour session including gay and lesbian physician panels, faculty member sharing his adjustment and affirmation of his child’s homosexuality, Q&A session	IAH administered at beginning of academic year, after initiative, end of academic year, and after clerkship	Decrease in homophobic attitude over time, though rebounding somewhat by the junior year	The group mean score remained in the low-grade homophobic category throughout the 3 year study Deeply held beliefs that influence patient care should be examined
